# Influence of local temperature on motor unit behavior during rapid contractions in humans

**DOI:** 10.1007/s00421-025-05796-0

**Published:** 2025-05-02

**Authors:** Kazutaka Ota, Hikaru Yokoyama, Kazushige Sasaki

**Affiliations:** 1 Department of Life Sciences, Graduate School of Arts and Sciences, The University of Tokyo, 3-8-1 Komaba, Meguro-ku, Tokyo, 153-8902 Japan; 2 Institute of Engineering, Tokyo University of Agriculture and Technology, 2-24-16 Naka-cho, Koganei-shi, Tokyo, 184-8588, Japan

**Keywords:** High-density surface electromyography decomposition, Rate of force development, Tibialis anterior, Explosive contraction

## Abstract

**Purpose:**

The rate of torque development (RTD) is temperature dependent, but the temperature effects on motor unit behavior during rapid contractions remain largely unknown. This study aimed to clarify the influence of local limb temperature on motor unit behavior and RTD during rapid contractions in humans.

**Methods:**

Ten healthy male participants rested in a sitting position while immersing their right lower leg in water at different temperatures (Hot: ~43 °C, Neutral: ~33 °C, and Cold: ~10 °C) for 20 min each. The participants then completed a series of voluntary isometric contractions of dorsiflexors while maintaining water immersion in each temperature condition. Specifically, they were instructed to perform two maximal voluntary contractions (MVC) followed by six rapid-hold contractions. High-density surface electromyography was recorded from the tibialis anterior muscle and decomposed into individual motor unit spike trains.

**Results:**

We found that the late RTD (from 0 to 150 ms after the torque onset) was significantly lower in Cold than in the other conditions even when normalized by MVC torque. The motor unit discharge rate at recruitment was significantly higher in Cold (51.4 ± 4.1 pps) than in Hot (42.0 ± 3.8 pps), while the recruitment threshold decreased with the temperature (Cold: 23.9 ± 2.7%, Neutral: 29.2 ± 2.5%, Hot: 36.2 ± 2.4% of MVC). The temperature-induced changes in the late RTD were significantly related to the changes in recruitment time and recruitment threshold.

**Conclusion:**

These findings suggest that local cooling induces earlier motor unit recruitment and higher discharge rate, mitigating the decrease in RTD.

**Graphical abstract:**

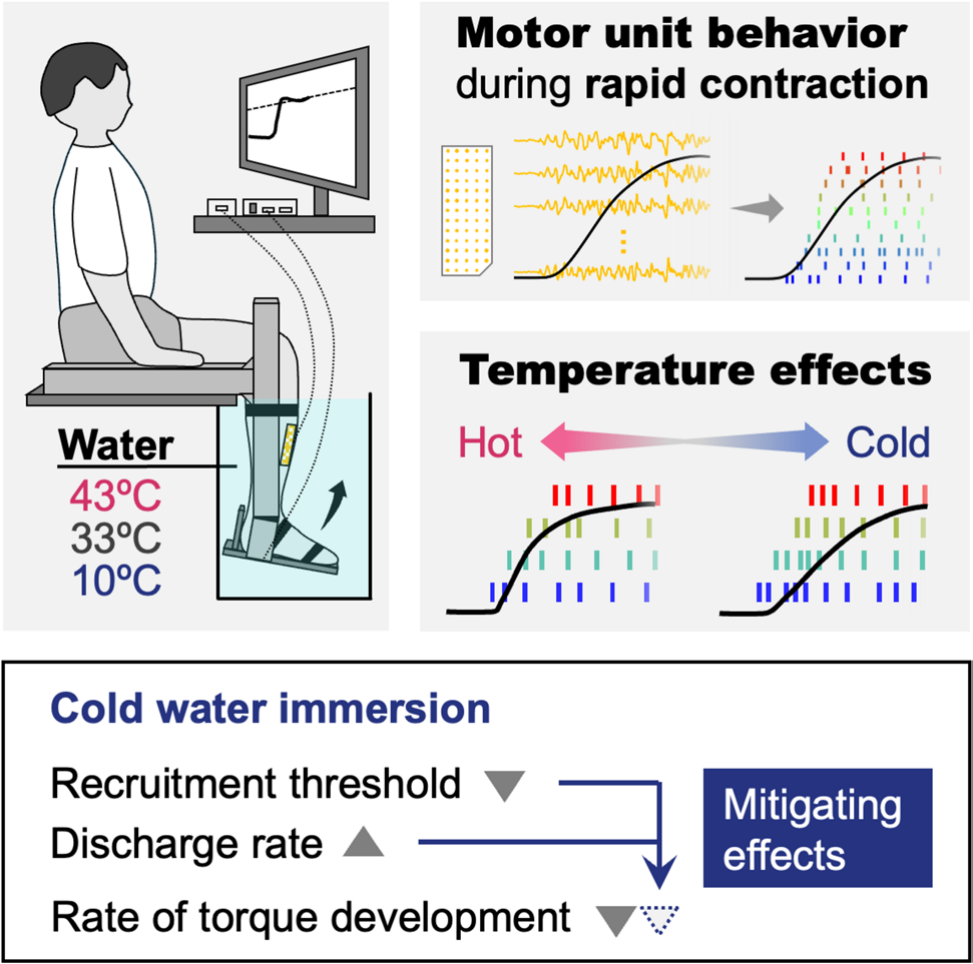

**Supplementary Information:**

The online version contains supplementary material available at 10.1007/s00421-025-05796-0.

## Introduction

Explosive muscle strength, the ability to produce high force in a brief period, is important in sports performance and injury prevention more than maximal, non-explosive muscle strength. This is because the time available for muscles to exert force during a jump, sprint, landing, and balance recovery upon a postural disturbance is under 250 ms (Do et al. 1982; Andersen and Aagaard 2006; Krosshaug et al. 2007), which is shorter than the time required to achieve maximal force (300 ms) (Thorstensson et al. 1976). The rate of torque development (RTD), rather than maximal strength or mechanical power, has been recognized as a key indicator of explosive athletic performance (Cronin and Sleivert 2005; Harris et al. 2007). Indeed, individuals with a higher RTD tended to achieve higher running speeds, whereas no significant correlation was found between maximal strength and running speed (Tillin et al. 2013a). Comparison of athletes (sprinters or jumpers) with untrained individuals showed a much larger difference in RTD during the first 50 ms of contraction than in maximal strength (Tillin et al. 2010). In addition, RTD is known to be associated with balance performance (Izquierdo et al. 1999), risk of falling (Pijnappels et al. 2008; LaRoche et al. 2010), and daily living functions such as speed of walking, stair climbing, and chair stands (Altubasi 2015; Osawa et al. 2018) among elderly individuals.

RTD is attributed to several muscular and neural determinants (Maffiuletti et al. 2016). The muscular determinants include muscle strength, size, architecture, fiber type composition, stiffness, and Ca^2+^ release rate (Maffiuletti et al. 2016). Regarding the neural determinants, it has long been established that neural activation at the onset of contraction, assessed by the amplitude of surface electromyography (EMG) signals, strongly influences RTD (De Ruiter et al. 1999; Tillin and Folland 2014). The advent of high-density surface EMG (HDsEMG) in recent years has provided convincing evidence that RTD is mainly determined by the motor unit recruitment speed and discharge rate at the initial phase of contractions (Del Vecchio et al. 2019; Dideriksen et al. 2020). According to previous research, the neural and muscular determinants contribute primarily to RTD in the early (< 75 ms) and late (> 75 ms) phases, respectively (Maffiuletti et al. 2016; Del Vecchio 2023). However, when the RTD is normalized to maximal strength, the neural determinants become dominant in both phases (Folland et al. 2014).

RTD is also influenced by some external factors such as auditory startling stimuli (Škarabot et al. 2022) and temperature (Cornwall 1994; Denton et al. 2016; Rodrigues et al. 2021). A decrease in the temperature of muscle and surrounding tissues (local temperature) reduces RTD during rapid contractions (Cornwall 1994; Denton et al. 2016). On the other hand, an increase in local temperature can enhance RTD (Denton et al. 2016; Rodrigues et al. 2021), but the effects are not as consistent as those of decreasing local temperature (Cornwall 1994).

There is no doubt that changes in RTD with temperature are strongly connected to changes in intrinsic contractile properties that can be inferred from the kinetics of twitch contraction (Davies et al. 1982; Davies and Young 1983; Mallette et al. 2019, 2021; Ota and Sasaki 2023). For instance, temperature-dependent decreases in the rate of Ca^2+^ release and reuptake from the sarcoplasmic reticulum result in the slowing of twitch tension development and relaxation (Rutkove 2001; Rodrigues et al. 2022). In contrast, studies on the relationship between temperature and motor unit behavior are limited to those using slow trapezoidal contractions (Mallette et al. 2018, 2021; Rodrigues et al. 2023). However, direct observation of motor unit behavior during rapid contractions is crucial because it is markedly different from those during slow contractions (Desmedt and Godaux 1977; Del Vecchio et al. 2019, 2022a). Although a previous study on knee extensors reported that skin cooling increased surface EMG amplitude during the early phase (0–100 ms) of rapid contractions (Shimose et al. 2014), it remains unexplored how individual motor unit behavior changes with local temperature during rapid contractions.

Therefore, this study aimed to explore the temperature dependence of motor unit behavior during rapid contractions in the human tibialis anterior muscle. Although there is no direct data on rapid contractions in humans, previous studies have raised the possibility that cooling induces additional recruitment of fast-twitch fibers or high-threshold motor units, which can mitigate the reduction in motor performance (Rome et al. 1984; Fujimoto et al. 2016; Mallette et al. 2018; Wakabayashi et al. 2018). Therefore, we hypothesized that decreasing local temperature would be associated with both a lower recruitment threshold and a higher discharge rate, mitigating the decrease in RTD.

## Methods

### Participants

Ten healthy, recreationally active male volunteers participated in this study. Their mean (SD) age, height, and body mass were 24.3 (1.1) years, 174.0 (4.2) cm, and 63.4 (5.7) kg, respectively. The volunteers were free from any neuromuscular disorder, lower limb pathology, or surgery. This study included only male participants because the number of motor units identified from HDsEMG signals is much lower in females than in males (Jenz et al. 2023). A priori sample size calculation was performed with G*Power version 3.1.9.6. (Heinrich Heine Universität Düsseldorf, Düsseldorf, Germany) using a within-participants analysis of variance (ANOVA) with a statistical power of 0.8 and an alpha error of 0.05. Seven participants were required to detect an effect size (Cohen’s *f*) of 0.6, which was based on a previous report on the effect of visual, auditory, and startling cues on motor unit behavior during rapid contractions (Škarabot et al. 2022). We increased the number to ten, considering other variables such as RTD during rapid contractions.

Before enrollment, written informed consent was obtained from each participant in accordance with the *Declaration of Helsinki* without being registered. This study was approved by the Ethical Review Committee for Experimental Research involving Human Subjects, Graduate School of Arts and Sciences, The University of Tokyo (Issue number: 1020).

### Overview of study

The present study used a within-participants design. Each participant visited our laboratory four times, with at least 48 h between visits. To reduce the potential impact of diurnal variations in limb temperature and motor performance, participants were instructed to keep their arrival times within 120 min of each other. All participants followed this instruction, except for one whose arrival times differed by up to 195 min because of personal circumstances. Consequently, the inter-day variation in arrival time was 47 ± 44 min (*n* = 10). The aim of the first visit was to familiarize participants with a series of maximal and submaximal voluntary isometric contractions of the right dorsiflexors. In the second to fourth visits, the participants completed a series of contractions with the right lower leg immersed in water at different temperatures. Specifically, we used water temperatures of ~43 °C (Hot), ~33 °C (Neutral), and ~10 °C (Cold). The order of temperature conditions was randomized across participants. Participants were asked to avoid any strenuous exercise (48 h) and caffeine and alcohol consumption (24 h) before visiting the laboratory.

### Experimental procedure

**Fig. 1:**
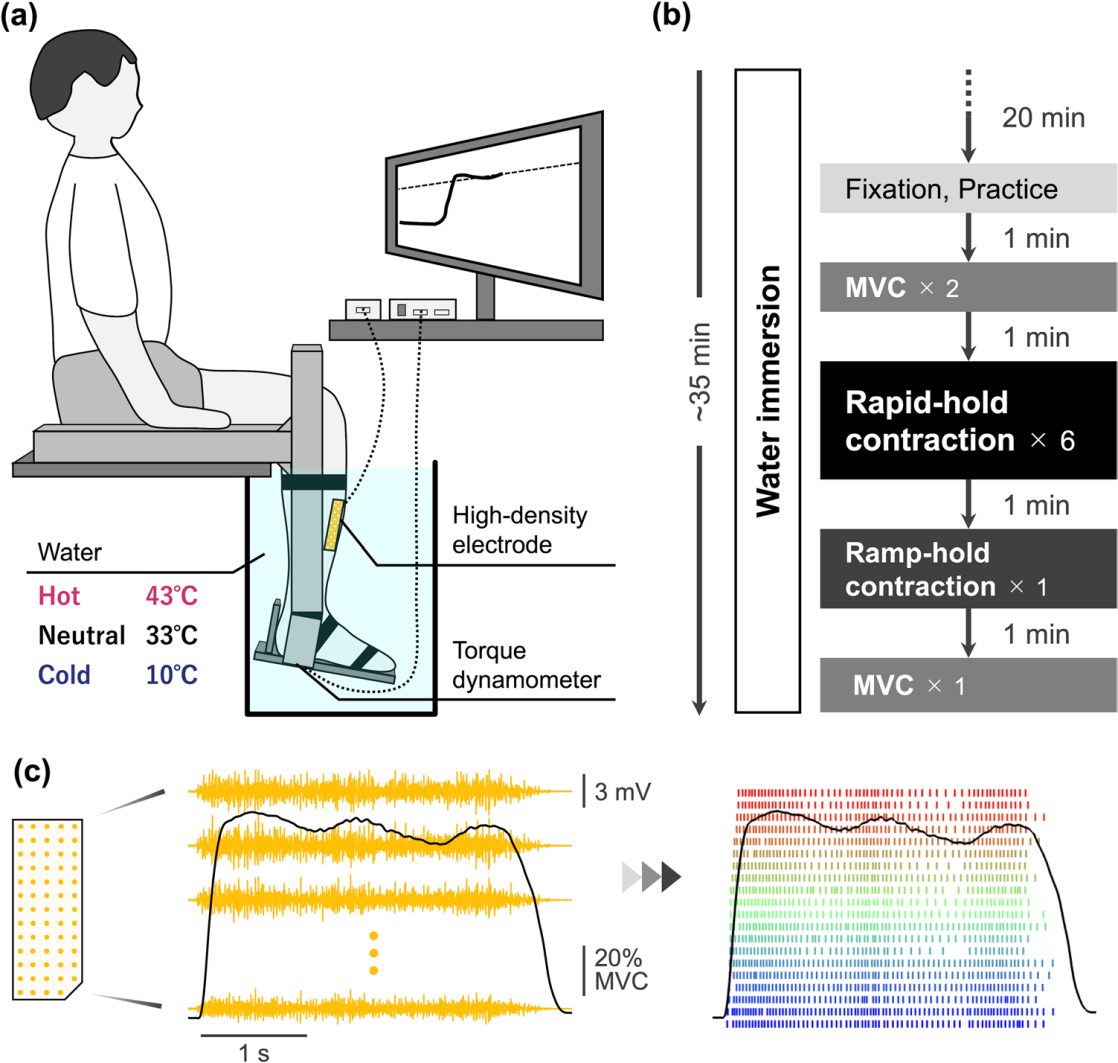
An overview of the experiment. **a** Experimental setup. Participants sat on an experimental chair, immersing their right lower leg in a large container filled with water at different temperatures. Dorsiflexion torque and high-density surface electromyography (EMG) signals from the tibialis anterior muscle were recorded. Participants received verbal encouragement and visual feedback of the torque signal. A target torque was displayed on the feedback screen of the exerted torque signal. **b** Experimental procedure. After 20 min from the initiation of water immersion, a series of contraction tasks were performed while maintaining the immersion. The participants performed three brief (~1 s) isometric dorsiflexions for practice. Next, they performed two 3-s maximal voluntary contractions (MVC). They then performed six rapid-hold contractions, with each contraction repeated every 20 s. Afterward, the participants performed a ramp-hold contraction. The experiment was closed with an additional MVC. **c** Typical recordings of dorsiflexion torque and tibialis anterior EMG signals during a rapid-hold contraction (left panel). The participants were instructed to produce joint torque as fast as possible to a level above 75% of MVC torque, and then to adjust and hold the torque at 75% MVC for 3 s. The EMG signals from 64 channels were decomposed into individual motor unit spike trains (right panel). Each row/color denotes discharges of an individual motor unit

After skin preparation and electrode placement for HDsEMG (see below), the right lower leg was immersed in a large container filled with water for 20 min in a sitting position (Fig. 1a). The temperature and duration of hot- and cold-water immersion were determined based on previous studies (Gossen et al. 2001; Ota and Sasaki 2023) and our pilot experiments (Online Resource 1), leading to a substantial change in twitch contractile function while minimizing pain, discomfort, and sweating. The water temperature was continuously monitored with a thermometer (Multi-thermometer, Japan Pet Design, Tokyo, Japan) and maintained within ± 1.0 °C of the target level by adding hot or cold water to the container until the end of experiment. During water immersion, the participants wore a slipper made of insulating material on their right foot and a plastic bag over the right lower leg to prevent the skin and electrodes from getting wet.

After 20 min from the initiation of water immersion, a series of contraction tasks were performed while maintaining the immersion (Fig. 1b). Each contraction was separated by a resting period of 1 min except for rapid-hold contractions (see below). After the fixation of the right lower leg to an ankle dynamometer, the participants performed three brief (~1 s) isometric dorsiflexions for practice. Next, the participants performed two 3-s maximal voluntary contractions (MVC). If the peak torque from the two trials differed by more than 5%, an additional trial was performed. The participants received verbal encouragement and visual feedback on the torque signal provided by data-recording software (LabChart 8, ADInstruments, Dunedin, New Zealand). The participants then performed six rapid-hold contractions, with each contraction repeated every 20 s in accordance with a recommendation (Maffiuletti et al. 2016). The participants were instructed to produce joint torque as fast as possible to a level above 75% of MVC torque, and then to adjust and hold the torque at 75% MVC for 3 s (Fig. 1c). The target torque was displayed as a horizontal line on the feedback screen of the exerted torque signal. The 3-s ‘hold’ phase was added to each rapid contraction to increase the number of identified motor units through HDsEMG decomposition (Del Vecchio et al. 2019). Afterward, the participants performed a ramp-hold contraction, where the participant linearly increased torque for 10 s from 0 to 20% MVC and maintained the target torque for 20 s. The experiment was closed with an additional MVC.

### Torque recording

A custom-designed ankle dynamometer was used to measure ankle joint torque produced by the dorsiflexor muscles. The participant rested in a sitting position with the right knee flexed at 90°. The right foot was fixed at an ankle joint angle of 100° (10° plantarflexion from the neutral position) and attached firmly to a footplate installed in the dynamometer using inelastic straps. The footplate was positioned so that its rotational axis coincided with the anatomical axis of the ankle. To prevent the tibia from moving forward during dorsiflexion, the knee was fixed to the dynamometer frame using an inelastic strap. The torque signal was obtained with strain gauges (KFGS-1–120-D16-11, Kyowa Electronic Instruments, Tokyo, Japan) attached to the rotational shaft of the footplate and digitized at a sampling rate of 2000 Hz using a data acquisition system (PowerLab C, ADInstruments).

### Torque analysis

Offline analysis was performed using MATLAB (R2023b, MathWorks, MA, USA). The torque signal during MVC was digitally low-pass filtered (zero-lag, sixth-order Butterworth filter) with a cutoff frequency of 20 Hz. The MVC torque was defined as the highest value in each trial. Of the two trials before a series of rapid-hold contractions, the one with the higher MVC torque was used for statistical analysis of MVC torque and global characteristics of EMG signals (see below). For the calculation of RTD during rapid-hold contractions, the onset of torque production was manually determined after the torque signal was digitally low-pass filtered (zero-lag, sixth-order Butterworth filter) with a cutoff frequency of 400 Hz (Tillin et al. 2010, 2013a). Torque signals were viewed with a constant *y* axis scale of ± 0.5 Nm and an *x* axis scale of 200 ms before the point at which the torque signal exceeded ~0.3 Nm. The torque onset was detected at the local minimum value before the signal deflected from the baseline. This detection method is considered more valid and accurate than existing methods using automated threshold detection (Tillin et al. 2013b). Following the determination of torque onset, the torque signal was digitally low-pass filtered (zero-lag, sixth-order Butterworth filter) with a cutoff frequency of 20 Hz. The contractions with no discernible countermovement or pretension (torque deflection of ± 0.1 Nm within 150 ms before the torque onset) were used for analysis. The early and late RTD were calculated by dividing the torque at 50 ms and 150 ms, respectively, after the torque onset by the corresponding time. The maximal RTD was calculated as the maximal value of RTD in overlapping time windows from 0–1 up to 0–250 ms (Del Vecchio et al. 2019, 2024).

### HDsEMG recording

HDsEMG signals were recorded from the tibialis anterior muscle. Following skin preparation including shaving, light abrasion, and cleaning with ethanol, a semi-disposable grid of 64 electrodes (13 rows, 5 columns, 1-mm electrode diameter, 8-mm inter-electrode distance; HD08MM1305, OT Bioelettronica, Torino, Italy) was placed over the proximal area. The electrode grid was attached to the skin with disposable bi-adhesive foam layers (FOA08MM1305, OT Bioelettronica), the cavities of which were filled with conductive paste (CC1, OT Bioelettronica) to guarantee good skin–electrode contact. To place the grid in an identical location and then identify the same motor units across visits (Del Vecchio et al. 2020), the position of the grid was marked on the skin by a permanent marker. A reference electrode (F-150, Nihon Koden, Tokyo, Japan) was placed on the tibial tuberosity of the right leg. HDsEMG signals were amplified in a monopolar configuration (10–500 Hz), sampled at 2000 Hz via a 16-bit wireless amplifier (Sessantaquattro+, OT Bioelettronica) using data acquisition software (OT Biolab+, OT Bioelettronica).

### HDsEMG analysis

Monopolar HDsEMG signals were filtered offline with notch (50 Hz, quality factor of 35) and band-pass (20–500 Hz, zero-lag, eighth-order Butterworth filter) filters. The filtered HDsEMG signals were decomposed into motor unit spike trains using an automatic convolutive blind source separation technique based on FastICA and the convolutional kernel compensation approach (Negro et al. 2016; Yokoyama et al. 2022). To increase the number of identified motor units during rapid-hold contractions, the HDsEMG signals obtained from the six rapid-hold contractions were concatenated with that from the ramp-hold contraction. The HDsEMG signals during the ramp-hold contraction were also decomposed independently. The quality of decomposition was evaluated using a silhouette value (Negro et al. 2016), which is defined as the difference between the within- and between-cluster sums of point-to-centroid distances, normalized by dividing by the larger value. In this study, motor units with a silhouette value of > 0.85 were used for further analysis (Murphy et al. 2019; Afsharipour et al. 2020; Yokoyama et al. 2021). Finally, physiologically irregular discharges were discarded. Specifically, discharges below 4 Hz were discarded from the rapid-hold contractions, while discharges below 4 Hz or above 50 Hz were discarded from the ramp-hold contraction (Adam and De Luca 2005; Hirono et al. 2022). Owing to changes in motor unit action potential waveforms with varying muscle temperatures (Mallette et al. 2018, 2021), motor units could not be tracked over the three temperature conditions.

As indicators of motor unit recruitment speed during rapid-hold contractions, we measured the time from the torque onset to the first discharge of each motor unit (recruitment time) and the torque at which a motor unit begins to discharge (recruitment threshold). However, we did not measure the number of motor units recruited per second (Del Vecchio et al. 2019; Škarabot et al. 2022) because it strongly depends on the number of identified motor units (Del Vecchio et al. 2022b). The motor unit discharge rate was defined as the average discharge rate over the first five discharges (Škarabot et al. 2022). Recruitment time, recruitment threshold, and discharge rate were averaged over three contractions with the highest late RTD to ensure maximal performance (Škarabot et al. 2022). The recruitment threshold and discharge rate were also calculated for the ramp-hold contraction, in which the discharge rate was averaged over a 15-s segment, excluding the first and last 2.5-s segments from a 20-s phase with a constant torque of 20% MVC. Motor units were included in the analysis only if they were recruited before the start of this segment and discharged at least five times within this segment.

To globally characterize the EMG signals during MVC, double differential signals were obtained from the monopolar signals approximately along the fiber direction (i.e., the long axis of the grid). The double differential signals were band-pass filtered (20–500 Hz, zero-lag, eighth-order Butterworth filter). The six signals with the highest cross-correlation in propagation were selected (Del Vecchio et al. 2017). We calculated the average of the root mean square amplitude and median frequency of the selected six signals over the 2-s segment that included the peak torque and had the smallest torque variability (i.e., the value obtained by dividing the SD by the mean). For the calulation of median frequency, fast Fourier transform with a Hamming window function was applied to a series of 256-ms segments (frequency resolution of 3.91 Hz) overlapping each other by 50% (Ida and Sasaki 2024).

### Statistics

The data are expressed as mean and SD. The analyses were carried out using R version 4.3.2 (R Foundation for Statistical Computing, Vienna, Austria) and the R packages “anovakun version 4.8.9 (Iseki 2025),” “lme4 (Bates et al. 2015),” “lmerTest (Kuznetsova et al. 2017),” and “emmeans (Lenth 2016).” The differences in RTD (both absolute and relative to MVC torque before rapid-hold contractions) and the number of identified motor units across the three temperature conditions were analyzed using a one-way analysis of variance (ANOVA) with repeated measures. The temperature effects on motor unit behavior (recruitment time, recruitment threshold, and discharge rate) were analyzed with a linear mixed model ANOVA (Aeles et al. 2023; Rodrigues et al. 2023; Hirono et al. 2024; Cabral et al. 2024) which included temperature condition as the fixed effect and participant as the random effect to account for individual variations in the number of identified motor units. For the torque and global EMG during MVC, a two-way (temperature and time) ANOVA with repeated measures was used to test whether the effect of time (the difference between before and after the series of rapid-hold contractions) was different between temperature conditions. When a significant main effect or interaction was found, post hoc multiple comparisons were conducted using *t* test, with the false discovery rate method used to correct *p* values (Benjamini and Hochberg 1995). Pearson’s product–moment correlation analysis was performed to examine associations of maximal RTD with early and late RTD. To demonstrate a relationship between temperature-induced changes in RTD and those in the individual average of motor unit behavior, a weighted regression analysis was performed with a linear mixed model that included temperature changes (Neutral to Cold and Neutral to Hot) and participants as the random effects. The data from each individual were weighted by the corresponding number of identified motor units averaged between Neutral and Cold, and between Neutral and Hot. Generalized *η*-squared (*η*_*G*_^*2*^), Cohen’s effect size for comparison between two mean values (*d*), Pearson’s product–moment correlation coefficient (*r*), and standardized beta coefficient (*β*) were reported as a measure of effect size for within-participant ANOVA, *t* test, correlation analysis, and weighted regression analysis, respectively. These effect sizes were interpreted as follows: *η*_*G*_^*2*^, ≥0.02 as small, ≥0.13 as medium, and ≥0.26 as large; *d*, ≥0.20 as small, ≥0.50 as medium, and ≥0.80 as large; *r* and *β*, ≥0.10 as small, ≥0.30 as medium, and ≥0.50 as large (Cohen 1988; Bakeman 2005; Lakens 2013). Adjusted conditional *R*-squared (*R*_*c*_^2^) was also reported for weighted regression analysis, reflecting the variance explained by both fixed and random effects (Nakagawa et al. 2017). A *p* value of <0.05 was considered statistically significant.

## Results

### Maximal voluntary contraction

Table 1 summarizes torque and global EMG data during MVC in each temperature condition. The two-way ANOVA and post hoc analysis revealed that MVC torque was lower in Cold than in Neutral (*d* = 0.405, *p* = 0.005), but EMG amplitude was not significantly affected by temperature. Both MVC torque and EMG amplitude decreased, but EMG frequency increased after the series of contraction tasks, as indicated by the main effects of time in the two-way ANOVA. However, the magnitude of these changes did not differ between temperature conditions, as revealed by no significant temperature-by-time interaction on MVC torque, EMG amplitude, or EMG frequency. The median frequency of the EMG signal was lower in Cold than in Hot (*d* = 1.463, *p* = 0.024) and Neutral (*d* = 1.444, *p* = 0.024).

**Table 1:** Cold water immersion decreased maximal voluntary contraction torque and frequency of global electromyographic signals

		Hot	Neutral	Cold	ANOVA		
					Factor	*η*_*G*_^*2*^ value	*p* value
Torque	Pre	46.8 (5.7)	48.9 (7.0)	45.4 (8.0)	**Temperature**	**0.031**	**0.049**
[Nm]	Post	44.3 (6.6)	46.3 (8.1)	43.6 (8.2)	**Time**	**0.024**	**0.008**
	Mean	45.5 (6.0)	47.6 (7.6)^*^	44.5 (8.0)	Temperature × Time	0.001	0.639
RMS	Pre	209 (121)	171 (65)	184 (68)	Temperature	0.029	0.554
[μV]	Post	172 (86)	147 (47)	166 (55)	**Time**	**0.031**	**0.016**
	Mean	190 (103)	159 (55)	175 (60)	Temperature × Time	0.003	0.239
MDF	Pre	113 (30)	112 (23)	83 (18)	**Temperature**	**0.276**	**0.004**
[Hz]	Post	133 (33)	121 (29)	94 (15)	**Time**	**0.068**	**0.029**
	Mean	123 (30)^*^	117 (24)^*^	88 (13)	Temperature × Time	0.009	0.178

Values are mean and SD (*n* = 10). Significant main effects in two-way ANOVA are highlighted in bold. Pre and Post indicate before and after a series of rapid-hold contractions, respectively. Average data of Pre and Post are also shown as Mean because no significant temperature-by-time interaction was found in the two-way ANOVA. Hot, Neutral, and Cold represent water immersion at ~ 43 °C, ~33 °C, and ~10 °C, respectively

*RMS* root-mean-square amplitude, *MDF* median frequency

^*^Significant difference from Cold, *p* < 0.05

### Rapid-hold contraction

**Fig. 2:**
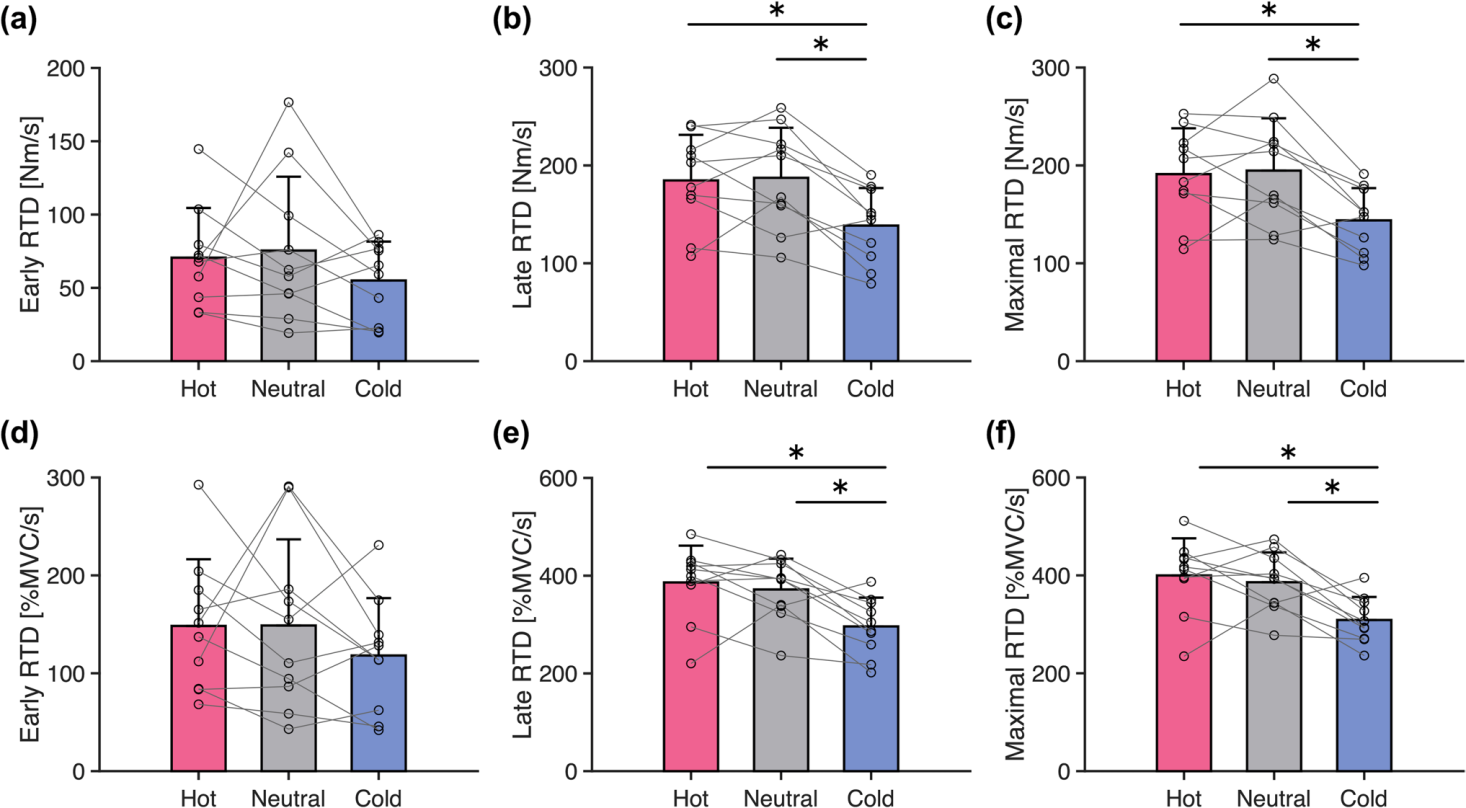
Cold water immersion decreased the rate of torque development (RTD) in the late phase of torque development. **a** RTD at 50 ms after the torque onset (early RTD). **b** RTD at 150 ms after the torque onset (late RTD). **c** Maximal value of RTD in overlapping time windows from 0–1 up to 0–250 ms (maximal RTD). **d** Early RTD relative to maximal voluntary contraction (MVC) torque. **e** Late RTD relative to MVC torque. **f** Maximal RTD relative to MVC torque. The data are presented as mean and SD with individual data points (*n* = 10). Hot, Neutral, and Cold represent water immersion at ~ 43 °C, ~33 °C, and ~10 °C, respectively. ^*^ *p* < 0.05

Figure 2 shows the differences in the rate of torque development (RTD) during the rapid-hold contractions. The absolute RTD at 50 ms after the torque onset (early RTD) was not significantly affected by temperature (one-way ANOVA, *η*_*G*_^*2*^ = 0.055, *p* = 0.303). Similarly, no significant temperature effect was found in the RTD relative to MVC torque (*η*_*G*_^*2*^ = 0.042, *p* = 0.383). Regarding the RTD at 150 ms after the torque onset (late RTD), the temperature effects were found on both absolute values (*η*_*G*_^*2*^ = 0.211, *p* < 0.001) and relative values (*η*_*G*_^*2*^ = 0.283, *p* < 0.001). The post hoc analysis revealed that the absolute and relative RTD were smaller in Cold than in Hot (absolute, *d* = 1.137, *p* = 0.002; relative, *d* = 1.399, *p* = 0.003) and Neutral (absolute, *d* = 1.139, *p* = 0.002; relative, *d* = 1.309, *p* = 0.003). Similarly, the temperature effects were found on the maximal RTD (absolute, *η*_*G*_^*2*^ = 0.226, *p* < 0.001; relative, *η*_*G*_^*2*^ = 0.313, *p* < 0.001) and the time from the torque onset to the maximal RTD (*η*_*G*_^*2*^ = 0.174, *p* = 0.001). The RTD was smaller in Cold than in Hot (absolute, *d* = 1.229, *p* = 0.002; relative, *d* = 1.517, *p* = 0.005) and Neutral (absolute, *d* = 1.208, *p* = 0.002; relative, *d* = 1.494, *p* = 0.005). The time to maximal RTD was longer in Cold (173.8 ± 32.3 ms) than in Hot (146.2 ± 24.7 ms, *d* = 1.011, *p* = 0.008) and Neutral (147.0 ± 30.7 ms, *d* = 0.895, *p* = 0.008). Maximal RTD was correlated with early RTD (*r* = 0.787, *p* < 0.001) and late RTD (*r* = 0.983, *p* < 0.001).

**Fig. 3:**
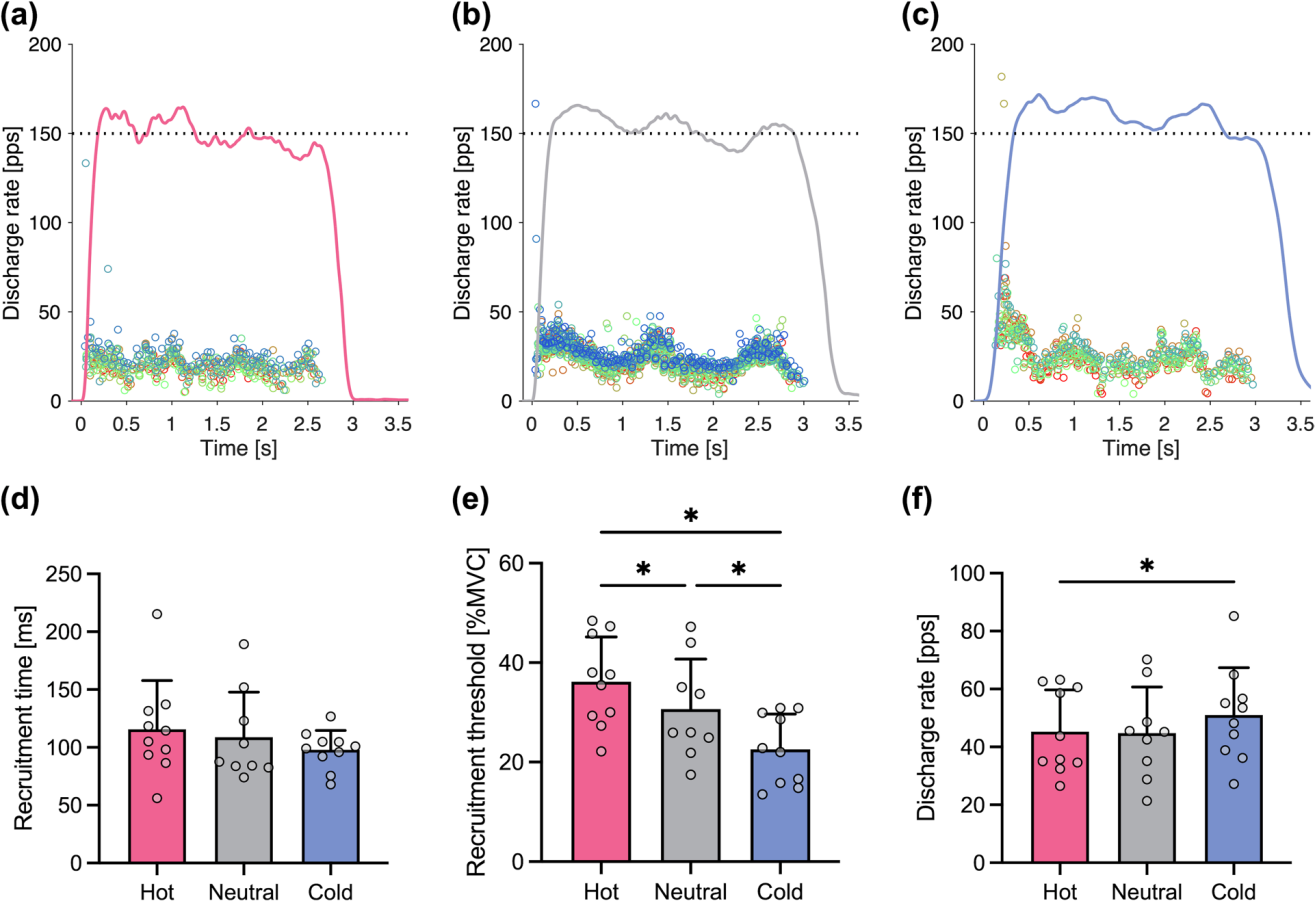
Cold water immersion decreased the motor unit recruitment threshold and increased the discharge rate. The upper panels show typical examples of each motor unit behavior (indicated by different colors) during the rapid-hold contractions at three different temperatures. Hot (**a**), Neutral (**b**), and Cold (**c**) represent water immersion at ~43 °C, ~33 °C, and ~10 °C, respectively. The red (**a**), gray (**b**), and blue (**c**) curves indicate the exerted torque, and the black dotted lines represent the reference torque corresponding to 75% of maximal voluntary contraction (MVC). **d** The temperature difference in the time from the torque onset to the first discharge of each motor unit (recruitment time). **e** The temperature difference in the relative torque at which each motor unit began to discharge (recruitment threshold). **f** The temperature difference in the discharge rate averaged over the first five discharges. In (**d**), (**e**), and (**f**), data are presented as mean and SD with individual data points (*n* = 10 except for Neutral, where no motor unit was identified in one participant). For clarity, each data point represents the average of all motor units identified from the same participant, while the statistical analysis was performed on individual motor unit data. ^*^ *p* < 0.05

**Fig. 4:**
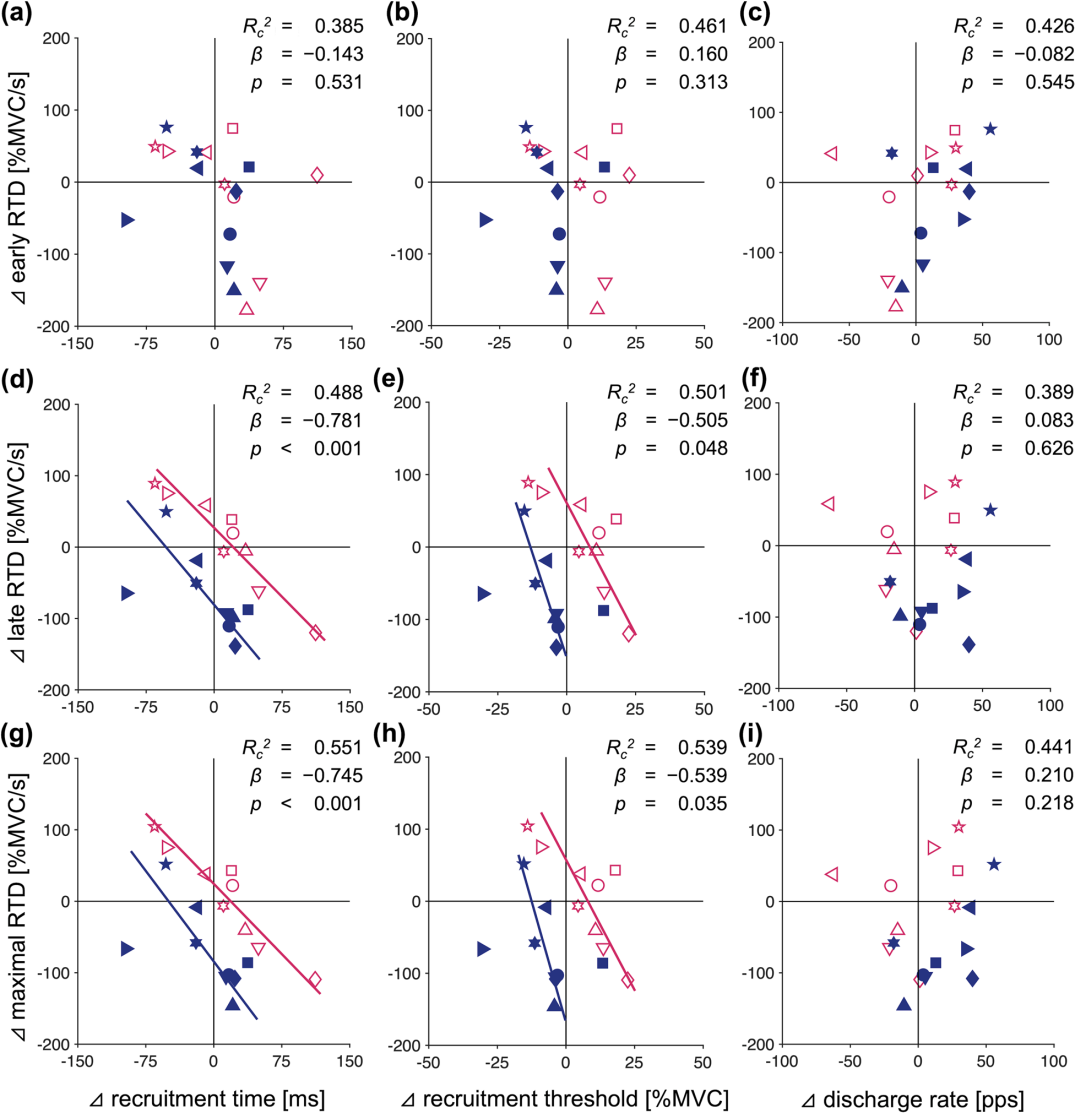
Temperature-induced changes in the rate of torque development (RTD) in the late phase of torque development were negatively associated with those in motor unit recruitment time and threshold. The upper panels show the associations of the changes in RTD at 50 ms after the torque onset (early RTD) with those in the individual average of recruitment time (**a**), recruitment threshold (**b**), and discharge rate (**c**). The middle panels show the associations of the changes in RTD at 150 ms after the torque onset (late RTD) with those in the individual average of recruitment time (**d**), recruitment threshold (**e**), and discharge rate (**f**). The lower panels show the associations of the changes in maximal value of RTD in overlapping time windows from 0–1 up to 0–250 ms (maximal RTD) with those in the individual average of recruitment time (**g**), recruitment threshold (**h**), and discharge rate (**i**). In each panel, adjusted conditional *R*-squared (*R*_*c*_^2^), standardized beta coefficient (*β*), and *p* value of weighted regression analysis with a linear mixed model are shown on the upper right corner. Each data point represents the change during water immersion at ~43 °C (red open symbols) and ~10 °C (blue filled symbols) when compared with a neutral temperature (~33 °C). Different symbols represent different participants. One participant was excluded from the analysis because of no identified motor unit at ~33 °C (thus *n* = 9 in each temperature for each panel). The weighted regression analysis revealed that temperature-induced changes in late and maximal RTD were significantly related to those in recruitment time (**d** and **g**, solid lines) and recruitment threshold (**e** and **h**, solid lines). Note that the relatively constant *R*_*c*_^2^ values across panels indicate the consistent contribution of random effects in explaining the variability of the RTD changes

A total of 204 motor units were identified from HDsEMG signals during the rapid-hold contractions. The number of identified motor units per participant during the rapid-hold contractions was unaffected by temperature (Table 2), as indicated by no significant main effect in the one-way ANOVA. Figure 3 shows individual and group data of motor unit behavior during the rapid-hold contractions at three different temperatures. The linear mixed model ANOVA revealed no significant main effect of temperature on recruitment time (*η*_*G*_^*2*^ = 0.012, *p* = 0.223) but on recruitment threshold (*η*_*G*_^*2*^ = 0.096, *p* < 0.001) and discharge rate (*η*_*G*_^*2*^ = 0.030, *p* = 0.029). The recruitment threshold was higher in Hot (*d* = 0.512, *p* = 0.003) and lower in Cold (*d* = 0.389, *p* = 0.034) than in Neutral. The discharge rate was higher in Cold than in Hot (*d* = 0.495, *p* = 0.026). The weighted regression analysis with the linear mixed model revealed that cooling-induced changes in late and maximal RTD were negatively related to those in recruitment time and recruitment threshold (Fig. 4). Specifically, individuals showing greater decreases in recruitment time and recruitment threshold exhibited a smaller decrease in late and maximal RTD with cooling, and vice versa. Similar relationships shifted to the upper right were observed in Hot condition, reflecting significant increases in RTD and recruitment threshold when compared with Cold condition.

**Table 2:** The number of identified motor units per participant was unaffected by temperature

	Hot	Neutral	Cold	ANOVA	
				*η*_*G*_^*2*^ value	*p* value
Rapid-hold	7.5 (3.9)	7.8 (5.5)	5.1 (2.9)	0.076	0.132
Ramp-hold	17.7 (8.0)	16.5 (7.8)	18.7 (7.3)	0.010	0.761

Values are mean and SD (*n* = 10). One-way ANOVA results are also shown. Hot, Neutral, and Cold represent water immersion at ~43 °C, ~33 °C, and ~10 °C, respectively

### Ramp-hold contraction

**Fig. 5:**
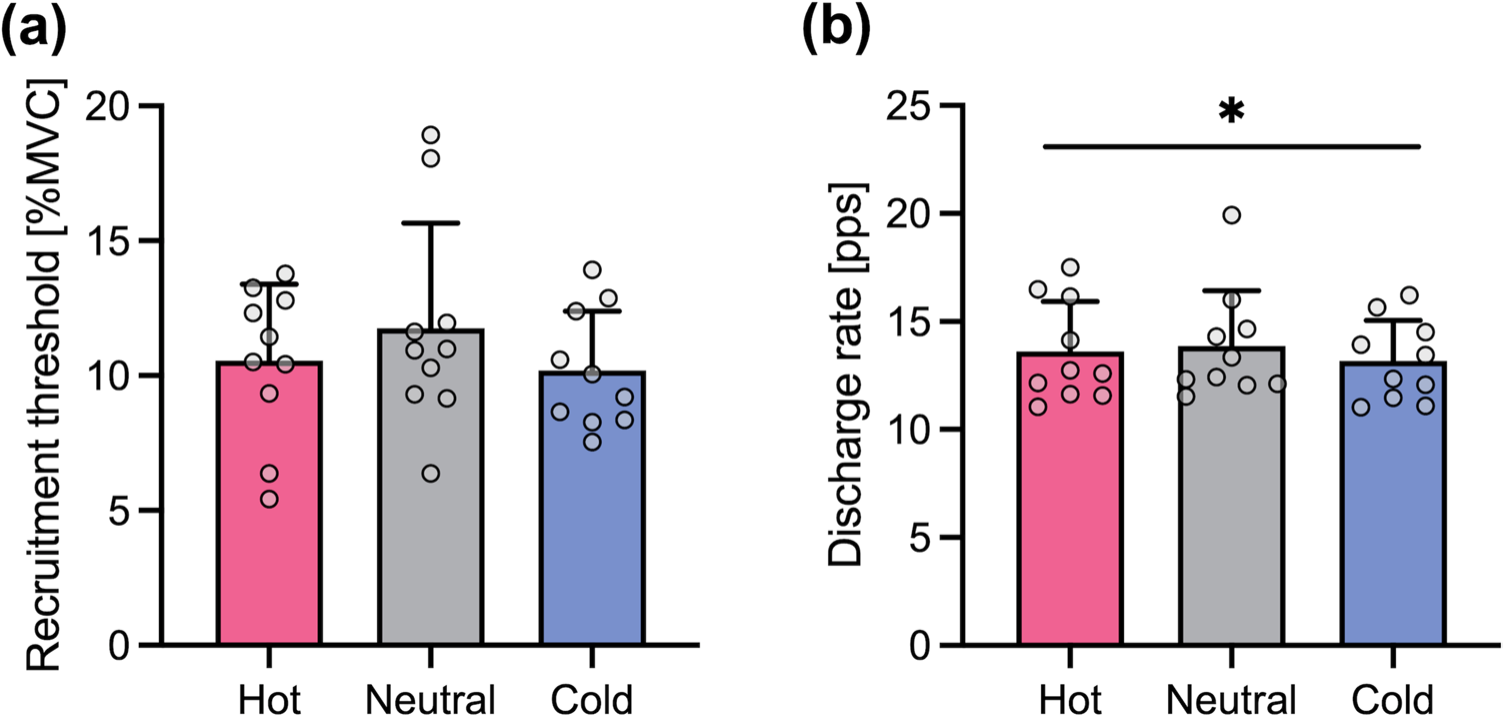
Cold water immersion decreased the motor unit discharge rate during a ramp-hold contraction. **a** The temperature difference in the relative torque at which a motor unit began to discharge (recruitment threshold). **b** The temperature difference in the discharge rate averaged over the torque plateau. Hot, Neutral, and Cold represent water immersion at ~43 °C, ~33 °C, and ~10 °C, respectively. The data are presented as mean and SD with individual data points (*n* = 10). For clarity, each data point represents the average of all motor units identified from the same participant, while the statistical analysis was performed on individual motor unit data. ^*^p < 0.05

A total of 525 motor units were identified from HDsEMG signals during the ramp-hold contraction. The number of identified motor units per participant during the ramp-hold contraction was unaffected by temperature (Table 2), as indicated by no significant main effect of temperature in the one-way ANOVA. Figure 5 shows the differences in motor unit behavior during the ramp-hold contraction. The linear mixed model ANOVA revealed no significant main effect of temperature on the recruitment threshold (*η*_*G*_^2^ = 0.005, *p* = 0.263), but on the discharge rate (*η*_*G*_^2^ = 0.009, *p* = 0.046). The discharge rate was higher in Hot than in Cold (*d* = 0.259, *p* = 0.047).

## Discussion

The temperature dependence of contractile properties is well documented in both animal and human muscles, but it remains elusive how neural drive and motor unit behavior during rapid contractions depend on temperature and consequently influence explosive performance, such as the rate of torque development (RTD). To our knowledge, the present study is the first to elucidate the influence of local temperature on the motor unit behavior during rapid contractions in humans. We hypothesized that decreasing local temperature would induce earlier motor unit recruitment and higher discharge rate, mitigating the decrease in RTD. As a support of this hypothesis, we found that local cooling decreased RTD, but changed motor unit behavior as if partially compensating for a loss of contractile performance. Indeed, the temperature-induced changes in motor unit behavior were linearly associated with those in RTD.

### Local cooling induced earlier motor unit recruitment and higher discharge rate

While the late RTD decreased with local cooling (Fig. 2), the motor unit recruitment threshold decreased, and the discharge rate increased (Fig. 3). Furthermore, there were significant associations between the temperature-induced changes in motor unit behavior and RTD (Fig. 4). Specifically, the smaller reduction in RTD was associated with the decreases in recruitment time and threshold, but not with the increase in discharge rate, which is consistent with the previous reports that RTD was more strongly associated with recruitment speed than discharge rate (Dideriksen et al. 2020; Del Vecchio et al. 2022a). These associations suggest that changes in motor unit behavior with local temperature act as a mitigating mechanism for alterations in explosive performance. Although we quantified RTD not only as those from specific time windows (early and late RTD) but also as the maximal RTD following a recent recommendation (Del Vecchio et al. 2024), the values of late and maximal RTD were similar (Fig. 2) and almost perfectly correlated (*r* = 0.982).

It should be noted that the decrease in recruitment threshold and the increase in discharge rate with local cooling were specific to the rapid-hold contractions. During a ramp-hold contraction, the recruitment threshold was not affected by temperature, and the discharge rate was higher in Hot than in Cold (Fig. 5). Motor unit behavior during ramp-hold contraction is dynamically regulated by afferent feedback from muscle (Bigland-Ritchie and Woods 1984) and assumed to be influenced by changes in both local temperature and muscle contractile performance. In contrast, motor unit behavior in the phase of torque development during rapid-hold contractions can be mainly characterized by feedforward neural drives (Del Vecchio et al. 2019) and therefore influenced by local temperature but not directly by temperature-dependent changes in contractile performance. Such a difference could contribute to the different impacts of temperature on motor unit behavior between ramp-hold and rapid-hold contractions.

The torque at which a motor unit begins to discharge, referred to as the ‘recruitment threshold,’ decreased with temperature, but the time from the torque onset to the first discharge of each motor unit, referred to as the ‘recruitment time,’ did not significantly differ across temperature conditions (Fig. 3). One might argue that the decline in the recruitment threshold due to local cooling merely reflects the reduction in RTD rather than representing the earlier recruitment. If this view is correct, temperature-induced changes in recruitment threshold should be positively associated with those in RTD. However, in each temperature condition where the confounding effect of intrinsic contractile properties is controlled, changes in both recruitment threshold and recruitment time were negatively associated with those in RTD (Fig. 4), which suggests that these changes have a mitigating effect on the changes in explosive performance.

### Potential explanation for temperature-induced changes in motor unit behavior

Several lines of evidence suggest that the cooling-induced decrease in recruitment threshold and increase in discharge rate can be attributed to the enhanced spinal excitability via the activation of cutaneous thermoreceptors. A previous study reported that whole-body skin cooling without changing core temperature enhanced the spinal excitability assessed with cervicomedullary evoked potentials (Hurrie et al. 2022). Another study reported that skin cooling with minimally affecting muscle temperature (~0.2 °C decrease) increased EMG amplitude during the early phase (0–100 ms) of rapid contractions (Shimose et al. 2014), suggesting the importance of cooling the skin rather than the muscle tissue. Indeed, transient receptor potential melastatin 8 (TRPM8), a major cold-sensing thermoreceptor, is much more expressed in free nerve endings in the skin than in muscle fibers (Schepers and Ringkamp 2009; Skagen et al. 2023). The TRPM8 activation by applying menthol to the skin was shown to increase EMG amplitudes during submaximal voluntary contractions (Tokunaga et al. 2017).

While local cooling resulted in a decrease in RTD, local heating did not significantly affect RTD (Fig. 2). This result is consistent with a previous report on human wrist extensors (Cornwall 1994) but not with more recent studies on leg muscles showing an increase in RTD by local heating (Denton et al. 2016; Rodrigues et al. 2021). The conflicting results can be explained by the differences in the muscle groups and the methods used to quantify RTD. We expect that the lack of change in RTD with local heating results from the combined effects (Gordon et al. 2021, 2023) of enhanced muscle contractile performance (Davies et al. 1982; Davies and Young 1983; Racinais et al. 2017; Mallette et al. 2019, 2021; Mornas et al. 2021; Gordon et al. 2021, 2023; Ota and Sasaki 2023) and reduced activation as evidenced by the higher recruitment threshold and the lower discharge rate of motor units (Fig. 3). One potential mechanism for the reduced activation with local heating is the hyperpolarization of the resting membrane potential. According to previous studies using the voltage–clamp technique (Kwieciński et al. 1984) and simulations (Stephanova and Daskalova 2014; Majumder et al. 2021), the resting membrane potential is hyperpolarized with heating and depolarized with cooling in humans. Alteration of the resting membrane potential could influence the motor unit behavior even when the amount of synaptic input is the same (Mallette et al. 2021).

### Methodological limitations

The present study has some methodological limitations that need to be considered. First, the number of identified motor units per participant per condition during rapid-hold contractions (~7) is small when compared with the total number of motor units in the tibialis anterior muscle (~200) (Duchateau and Enoka 2022). However, it should be noted that the small number of identified motor units is not specific to this study but an inherent limitation of HDsEMG technique (Del Vecchio et al. 2020). This technical limitation also led to the inclusion of only male participants (Škarabot et al. 2022), as the number of identified motor units is generally much lower in females than in males, probably owing to the larger amount of subcutaneous fat (Jenz et al. 2023). To account for individual variations in the number of identified motor units, we used a linear mixed model analysis (Aeles et al. 2023; Rodrigues et al. 2023; Hirono et al. 2024; Cabral et al. 2024) instead of traditional ANOVA based on the representative values of identified motor units in each participant and condition. In addition, the motor unit decomposition technique does not necessarily track the same motor units across the three temperature conditions because of the possible changes in action potential waveforms resulting from variations in conduction velocity (Mallette et al. 2018, 2021). Tracking the same motor units may be feasible by continuously recording HDsEMG while altering local temperature, similar to the way of tracking the same motor units under slowly changing joint angles (Yokoyama et al. 2021).

Second, we did not collect data on limb temperature or twitch contractile properties to avoid interrupting water immersion or causing an unexpected electric shock to the participant. However, we are confident that the changes in local temperature were large enough to induce changes in muscle contractile properties and motor unit behavior in this study. As mentioned in Methods, our water immersion protocol (e.g., temperature and duration) was established based on a pilot experiment (Online Resource 1) and previous findings of significant changes in muscle temperature and twitch contractile properties (Gossen et al. 2001; Ota and Sasaki 2023). Nevertheless, future studies are warranted to identify an interplay between temperature-induced changes in contractile properties and those in motor unit behavior.

## Conclusion

Immersing a lower leg in cold water resulted in a decrease in the late and maximal RTD during rapid isometric contractions of the dorsiflexors. Using the motor unit decomposition technique with HDsEMG, we also demonstrated that local cooling decreased the recruitment threshold and increased the discharge rate. The temperature-induced changes in the late and maximal RTD were significantly related to the changes in recruitment time and recruitment threshold. These findings suggest that local cooling induces earlier motor unit recruitment and higher discharge rate, mitigating the decrease in RTD. Future studies are warranted to explore the potential use of temperature-dependent motor unit behavior as a strategy to improve explosive and balance performance in both athletes and vulnerable populations.

## Supplementary Information

Below is the link to the electronic supplementary material.

Supplementary file1 (DOCX 6517 KB)

## Data Availability

Data will be made available upon reasonable request.

## Abbreviations

ANOVA: Analysis of variance
EMG: Electromyography
HDsEMG: High-density surface electromyography
MVC: Maximal voluntary contraction
RTD: Rate of torque development
TRPM8: Transient receptor potential melastatin 8
