# Supplementary material for: Influence of local temperature on motor unit behavior during rapid contractions in humans
Source: Eur J Appl Physiol. 2025 May 2;125(9):2581–95. doi: 10.1007/s00421-025-05796-0 (PMC12423161; doi:10.1007/s00421-025-05796-0)
Supplement: Supplementary file 1 — Supplementary file1 (DOCX 6517 KB) [file 421_2025_5796_MOESM1_ESM.docx]

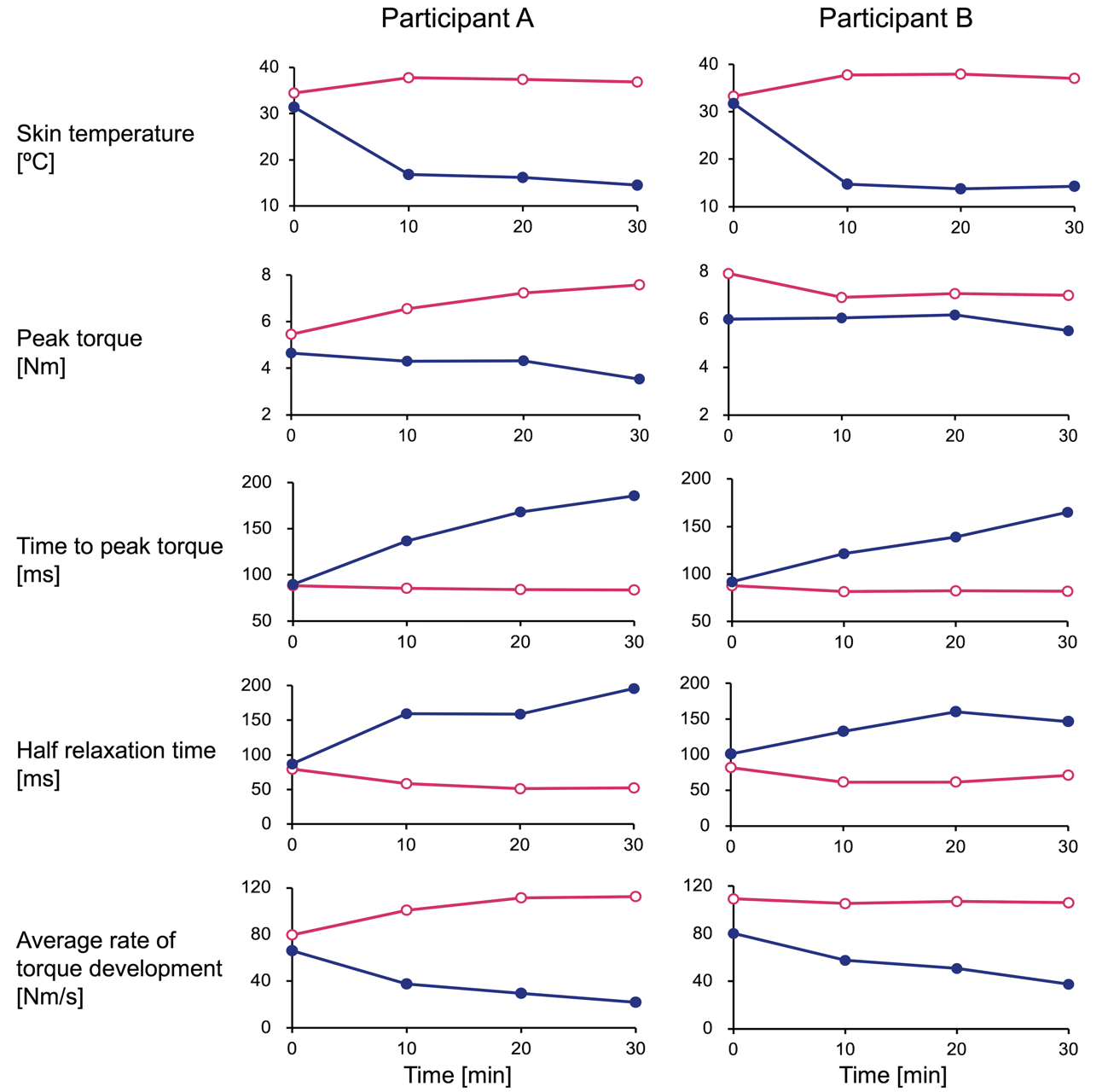


**Online Resource 1**

**Figure**. Changes in skin temperature and twitch contractile properties during water immersion at 43 °C (red open circles) and 10 °C (blue filled circles). Data from a subset of participants, namely participants A (left) and B (right), are shown. Measurements were completed within a brief period of time (up to 1 min) every 10 min from the beginning of immersion, during which the immersion was interrupted. Skin temperature was measured using an infrared thermometer (Thermofocus-pro, Technimed, Varese, Italy). Twitch contractions of dorsiflexor muscles were evoked with a supramaximal 0.5-ms rectangular pulse delivered by an electric stimulator (SEN-3401, Nihon Koden, Tokyo, Japan) with a stimulus-isolation unit (SS-2185, Nihon Koden) through two Ag–AgCl surface electrodes (F-150, Nihon Koden). The following twitch contractile properties were analyzed: the highest value of twitch torque (peak torque), the time from electrical stimulus to peak torque (time to peak torque), the time for peak torque to decay by 50% (half relaxation time), and the peak torque divided by the time from torque onset to peak torque (average rate of torque development).

**Influence of local temperature on motor unit behavior during rapid contractions in humans**

**European Journal of Applied Physiology**

Kazutaka Ota^1*^, Hikaru Yokoyama^2^, Kazushige Sasaki^1^

^1^ Department of Life Sciences, Graduate School of Arts and Sciences, The University of Tokyo, Tokyo, Japan

^2^ Institute of Engineering, Tokyo University of Agriculture and Technology, Tokyo, Japan

^*^ Corresponding author

Email: gotham1731@g.ecc.u-tokyo.ac.jp

ORCID: 0000-0001-6145-8566
